# Thermal conductivity enhancement in electrospun poly(vinyl alcohol) and poly(vinyl alcohol)/cellulose nanocrystal composite nanofibers

**DOI:** 10.1038/s41598-019-39825-8

**Published:** 2019-02-28

**Authors:** Yeongcheol Park, Myungil You, Jihoon Shin, Sumin Ha, Dukeun Kim, Min Haeng Heo, Junghyo Nah, Yoong Ahm Kim, Jae Hun Seol

**Affiliations:** 1 School of Mechanical Engineering, Gwangju Institute of Science and Technology (GIST), Buk-gu, Gwangju 61005 Korea; 2 Center for Environment & Sustainable Resources, Korea Research Institute of Chemical Technology (KRICT), 141 Gajeong-ro, Yuseong-gu, Daejeon 34114 Korea; 3 Department of Advanced Materials & Chemical Engineering, University of Science & Technology (UST), 217, Gajeong-ro, Yuseong-gu, Daejeon 34113 Korea; 4 Department of Polymer Engineering, Graduate School, Chonnam National University, 77, Yongbong-ro, Buk-gu, Gwangju 61186 Korea; 5 Smart Textile R&D team, Korea High Tech Textile Research Institute, 170, Geomjun-gil, Nam-myeon, Yangju-si, Gyeonggi-do 11410 Korea; 6 Department of Electrical Engineering, Chungnam University, Yuseong-gu, Daejeon 34134 Korea; 7 Present Address: Electric Power Conversion System Engineering Design Team, Hyundai Motor Group, 150, Hyundaiyeonguso-ro, Namyang-eup, Hwaseong-si, Gyeonggi-do 18280 Korea

## Abstract

The thermal conductivity enhancement of neat poly(vinyl alcohol) and poly(vinyl alcohol) (PVA)/cellulose nanocrystal (CNC) composite was attempted via electrospinning. The suspended microdevice technique was applied to measure the thermal conductivity of electrospun nanofibers (NFs). Neat PVA NFs and PVA/CNC NFs with a diameter of approximately 200 nm showed thermal conductivities of 1.23 and 0.74 W/m-K, respectively, at room temperature, which are higher than that of bulk PVA by factors of 6 and 3.5, respectively. Material characterization by Fourier transform infrared spectroscopy, differential scanning calorimetry, and thermogravimetric analysis confirmed that the thermal conductivity of the PVA/CNC NFs was enhanced by the reinforcement of their backbone rigidity, while that of the neat PVA NFs was attributed to the increase in their crystallinity that occurred during the electrospinning.

## Introduction

Polymer nanofibers (NFs) have been developed for a variety of applications due to their high surface volume ratios, flexible functionalities, and outstanding mechanical properties^1^. Among various NF synthesis methods, electrospinning has attracted great interest because it produces fiber-constituting structures, such as a micro- or nanoscale porous membranes from various polymers, and it is suitable for mass production^1,2^. Therefore, electrospun NFs have been used for filters^3,4^, tissue templates^5,6^, protective clothing^7,8^, and so forth. Among more than fifty different electrospinnable polymers, poly(vinyl alcohol) (PVA)-based NFs show great potential for use in novel biomedical applications, such as tissue scaffolding and wound dressing, due to their water solubility, biocompatibility, and biodegradability^1^.

The properties of PVA-based electrospun NFs can be reinforced by adding nanoscale fillers as those of various other polymer composites. Several reports have been published regarding reinforcement of the mechanical properties of electrospun NFs with nanoscale fillers, such as carbon nanotubes^9,10^, graphene^11^ and cellulose nanocrystals (CNCs)^12,13^. However, the mechanism of thermal conductivity (*k*) change in an electrospun NF with nanoscale fillers has not been fully elucidated yet. Interestingly, there are two critical factors simultaneously involved in thermal transport in such materials, namely, molecular chain alignment via electrospinning and the effect of fillers on *k* enhancement.

Generally, the thermal conductivity of polymers ranges from 0.1 to 0.5 W/m-K^14^. Such low *k* is ascribed to the amorphous aspects of polymers, that is, their randomly oriented structure and entangled molecular chains^15^. Therefore, the polymer *k* is strongly affected by the crystallinity of polymers: polymers with higher crystallinities possessed higher *k* values than those with lower crystallinities^16,17^. For instance, the *k* value of highly oriented polyethylene is higher than that of an amorphous polyethylene by a factor of twenty-five^16^. Recently, a *k* value of approximately 104 W/m-K was obtained from an ultra-drawn polyethylene NF^18^. Successively, more than 20-fold enhancements of *k* values were reported for electrospun and rapidly stretched polyethylene NFs^19,20^. In addition to polyethylene NFs, the *k* values of Nylon-11, polystyrene, and epoxy resin NFs were also significantly higher than those of their bulk counterparts^21–23^. This dramatic *k* increase proposes a possibility that polymer nanofibers can be used for various thermal applications such as thermal interface and functional fabric materials.

Although the *k* values of electrospun NFs were significantly enhanced, the role of nanoscale fillers in electrospun composite NFs is not fully understood yet. The thermal conductivity of an electrospun composite NF is expected to increase because the addition of high *k* fillers (*e*.*g*., metallic fillers and carbon nanotubes) is one of the representative methods to improve the thermal conductivities of polymers^24^. In addition to *k* enhancement by high *k* fillers (*i*.*e*., the effective medium theory) there could be another factor that affects the thermal conductivity of an electrospun composite NF depending on the interaction between the matrix polymer and the nanoscale fillers. For instance, numerous studies have reported that the mechanical properties were improved when matrix polymers and nanoscale fillers formed hydrogen bond networks^12,25,26^. Among these studies, Peresin *et al*. reported that enhancement of the elastic modulus in electrospun PVA/CNC NFs was observed, which was attributed to CNC reinforcement^12^. Uddin *et al*. observed that the tensile strength and the toughness of a PVA/CNC composite were maximized at 5% (w/w) of CNC in the composite^25^. As Young’s modulus (*E*) is related to *k*, *e*.*g*., $k\propto \sqrt{E}$, the thermal conductivity of an electrospun composite NF would be influenced by the interaction between the matrix polymer and nanoscale fillers.

In the present study, we measured the thermal conductivities of CNC-filled PVA electrospun fibers. Cellulose nanocrystal has recently attracted considerable attention because it is a biodegradable, renewable, and mechanically superb material^27^. Moreover, CNC also possesses plentiful hydroxyl (–OH) groups; thus, it forms hydrogen bonds with the PVA matrix. We attempted to elucidate the role of rigid fillers, *i*.*e*., CNCs, as well as that of molecular chain alignment in the *k* enhancement of PVA/CNC composite NFs by conducting *k* measurements and material characterizations, such as Fourier transform infrared spectroscopy (FTIR), differential scanning calorimetry (DSC), and thermogravimetric analysis (TGA).

## Results and Discussion

**Figure 1:**
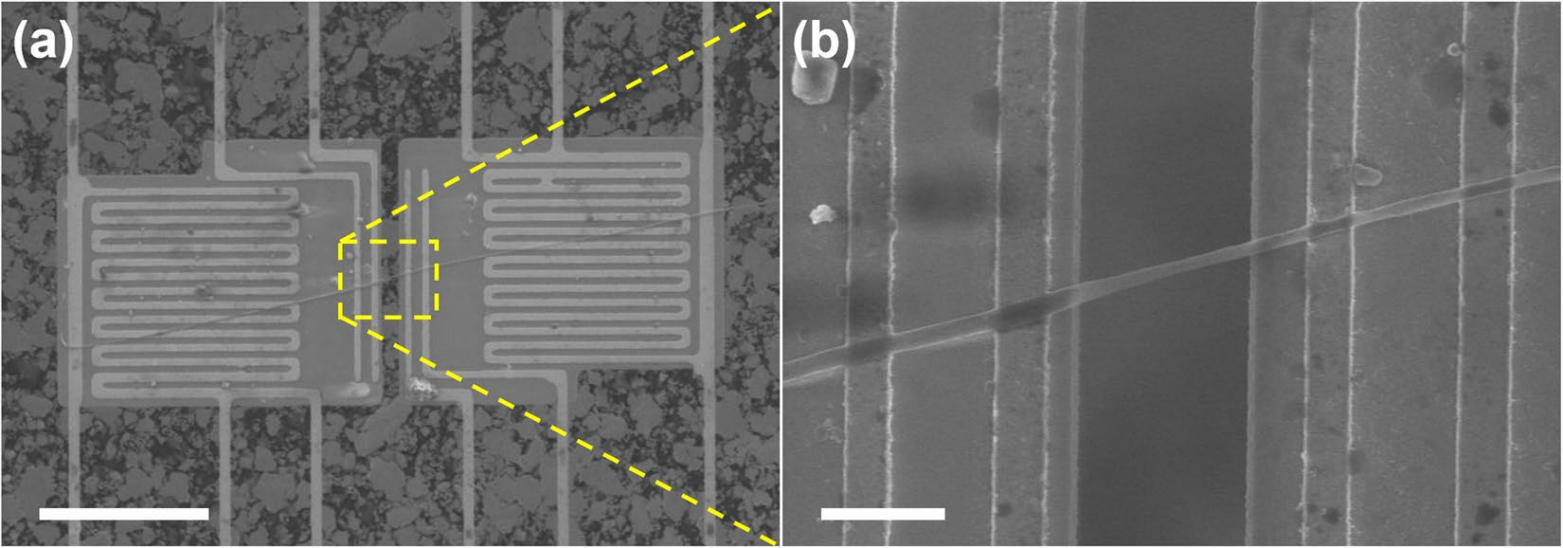
SEM images of an electrospun neat PVA NF assembled on a suspended microdevice. The whole region of the suspended membranes and the NF are included in (**a**). The suspended segment of the NF is enlarged in (**b**). To improve the thermal contacts between the NF and the suspended membrane, a drop of non-polar solution was released on the suspended microdevice with the NF after laser cutting. The scale bars are 25 μm and 2 μm, respectively.

To measure the thermal conductivity of PVA and PVA/CNC NFs, nine NFs with different CNC contents of 0, 2, and 5% (w/w) were assembled on suspended microdevices as shown in Fig. 1 (for details see Methods). There were three NF samples prepared for each CNC content, respectively, and the NFs were named considering their CNC contents (see Table S1). The diameters of the assembled NFs ranged from 120 to 411 nm.

**Figure 2:**
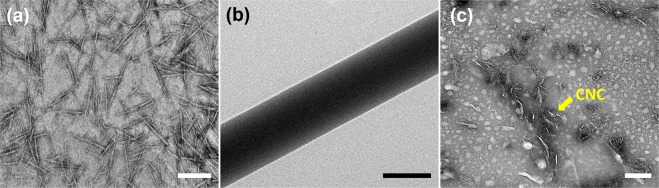
TEM images of cellulose nanocrystals isolated from (**a**) microcrystalline cellulose (MCC), (**b**) neat PVA NFs, and (**c**) the CNC dispersion of PVA/CNC-2% (w/w) NFs. The scale bars in (**a**–**c**) are 200 nm. The length of CNCs is approximately 300 nm. The bright dots in Fig. 2c formed when water on the microtomed section evaporated.

The dimensions of the CNCs were determined by TEM image analysis, as shown in Fig. 2a, resulting in a width of 7 ± 2 nm and a length of 300 ± 100 nm. TEM images of electrospun NFs were taken as shown in Fig. 2b and the Supplementary Information. Additionally, the homogeneous dispersion of CNCs in PVA/CNC NFs was verified via TEM as shown in Fig. 2c. Cellulose nanocrystals became discernable when they were located in the microtome slice plane and thus exposed to uranyl acetate. Figure 2c also confirms that the length of the CNCs, of which the axes are parallel to the microtome slice plane, is approximately 300 nm.

**Figure 3:**
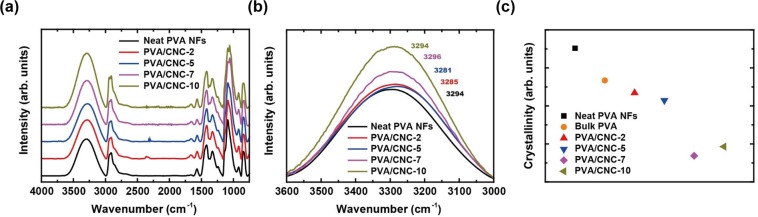
Fourier transform infrared spectra (FTIR) of electrospun neat PVA and PVA/CNC NFs (**a**) in the full wavenumber range of 750 to 4000 cm^−1^ and (**b**) in the range of 3000 to 3600 cm^−1^, which are involved in the –OH group vibrations. (**c**) The crystallinity values of neat PVA NFs and PVA/CNC NFs, which were obtained from the peak intensity ratios of 1142 to 1094 cm^−1^. Also, the crystallinity of bulk PVA^28^ was added in the figure for comparison.

**Figure 4:**
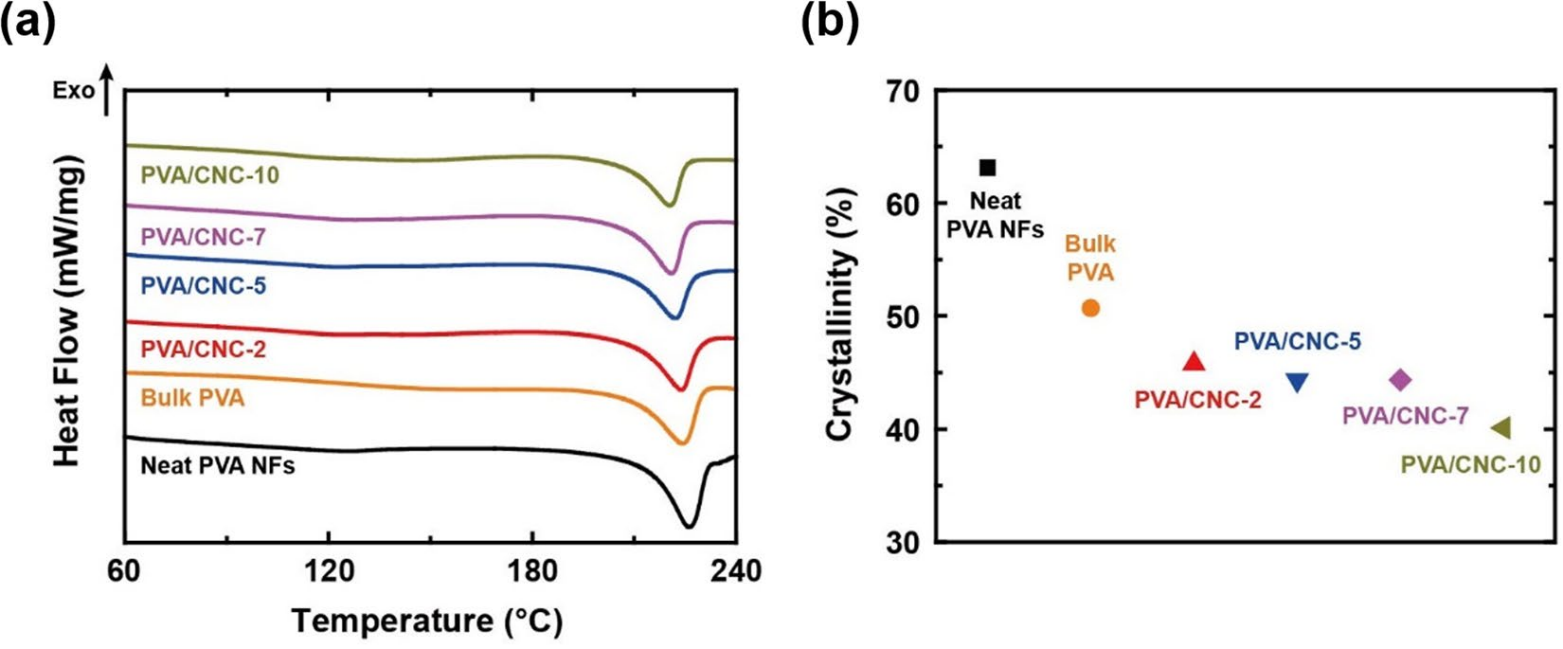
(**a**) Differential scanning calorimetry (DSC) curves and (**b**) the degree of crystallinity of neat PVA NFs, bulk PVA, and PVA/CNC NFs. The melting temperature and crystallinity of the PVA/CNC NFs were lower than those of neat PVA NFs and bulk PVA because the chain alignment of PVA was restricted by the formation of hydrogen bonds with CNCs^12,29^.

Regarding the thermal conductivity of NFs, there are two crucial factors, namely, molecular chain alignment and hydrogen bonding, which need further investigation. In this study, the degree of molecular chain alignment was quantified from FTIR measurements based on the characteristic that the crystallinity of PVA was proportional to the ratio of peak intensity at 1142 cm^−1^ to that at 1094 cm^−1^^28^. Although X-ray diffraction is a more reliable method for this purpose, it is difficult to apply this method due to the random alignment of electrospun NFs. As shown in Fig. 3c, the peak intensities at 1142 and 1094 cm^−1^ are involved in the degree of crystallinity and the C–O stretching vibration, respectively. The neat PVA NFs had the highest crystallinity and the PVA/CNC NFs had lower crystallinity than bulk PVA because PVA is a semi-crystalline polymer^28^. The crystallinity of bulk PVA originates from 10- to 20-nm-thick plate-like crystalline structures, *i*.*e*., lamellae^28^. The highest crystallinity of the neat PVA NFs can be ascribed to the enhanced molecular chain alignment resulting from the influence of shear stress during electrospinning. Also, disorder, which is induced by the addition of fillers, reduces the crystallinity in the composite NFs^12,29,30^. As opposed to our expectation, the crystallinity of PVA/CNC-7% (w/w) NFs was slightly lower than that of PVA/CNC-10% (w/w) NFs, which might be due to the uncertainty of data reduction from the FTIR measurement (Fig. 3c). For instance, as the CNC content of the PVA/CNC NFs increased above 7%, it was difficult to differentiate the peaks at 1142 and 1094 cm^−1^ via deconvolution due to the protrusion of the C–OH stretching peaks of CNC at 1060 (secondary –OH) and 1035 cm^−1^ (primary –OH)^29,31^ (Supplementary Information). Therefore, the FTIR analysis could only confirm that the crystallinity of neat PVA NFs was higher than that of the PVA/CNC NFs. Alternatively, DSC measurements were performed to quantify the crystallinity with respect to CNC content. As shown in Fig. 4a, the endothermic peaks exhibited a shift of the melting temperature (*T*_m_) from 226 to 220 °C with an increase of the CNC content to 10%. The DSC characterization indicates that the crystallinity of bulk PVA was lower than that of neat PVA NFs and higher than that of the PVA/CNC NFs (Fig. 4b). Hereafter, PVA/CNC-2% (w/w) and PVA/CNC-5% (w/w) are referred to as PVA/CNC-2 and PVA/CNC-5, respectively.

The possibility that hydrogen bonds directly increase *k* was also considered. The contribution of hydrogen bonds to thermal transport has been thought to be negligible, taking into account that hydrogen bonds are approximately 100 times weaker than covalent bonds. On the contrary, an interesting result was observed in polymer blends, of which the *k* value was as high as 1.5 W/m-K^32^. The high thermal conductivity of the polymer blends can be attributed to comparatively strong hydrogen bonds with short lengths. Although anhydroglucose ring oxygens or –OH groups in CNCs participate in the formation of bonds with –OH groups in the side chains of PVA^33^, the total number of hydrogen bonds between PVA molecules could be decreased by the physical barrier effect of CNCs, which disturb the formation of hydrogen bonds between PVA molecular chains^12,29,30^. In this study, the reduction of hydrogen bonds by the disturbance of CNCs was observed in the spectra of PVA/CNC-2 and PVA/CNC-5 NFs, judging from the slight peak position decrease of the –OH group band in the PVA/CNC spectrum^34^ (Fig. 3b). However, such a peak position decrease did not appear in the spectra of the PVA/CNC-7 and PVA/CNC-10 NFs. Additionally, the difference between the spectra of the PVA/CNC NFs and neat PVA NFs showed a significant change in –OH frequencies of 3000 to 3600 cm^−1^ as Peresin *et al*. observed (Supplementary Information)^12^. Hydrogen-bonded –OH and free –OH groups produce peaks at relatively lower and higher frequencies, receptively. Thus, the shape of the subtracted spectra (subtracting the spectrum of the neat PVA NFs from that of the PVA/CNC NFs) can provide information regarding the status of hydrogen bonds in the PVA/CNC NFs. As shown in Fig. S6, the PVA/CNC-2 and PVA/CNC-5 NFs have two different peaks in the –OH band, which can be interpreted as increases in both hydrogen-bonded –OH and free –OH groups. As the CNC content increases, the peaks are amplified further and seem to merge. The –OH band in the difference spectra shows the interaction between CNCs and PVA molecules through hydrogen bonds.

**Figure 5:**
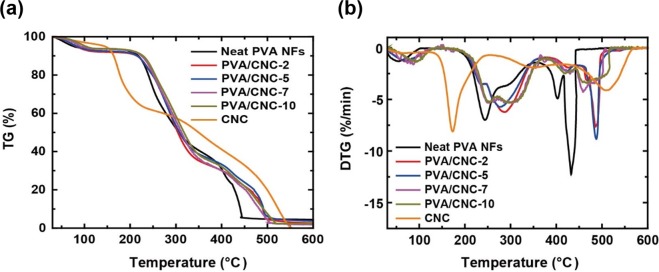
(**a**) Thermogravimetric analysis (TGA) curves of neat PVA NFs, CNCs, and PVA/CNC NFs and (**b**) corresponding derivative TGA curves. The major weight losses of the neat PVA NFs were observed in the range of 200 to 350 and 380 to 440 °C, respectively, which originated from the dehydration of PVA molecular chains and chain scission, respectively. Compared with the neat PVA NFs, the PVA/CNC NFs had higher weight loss temperatures by approximately 285 and 485 °C for dehydration and chain scission, respectively, due to their enhanced thermal stability. Cellulose nanocrystals were mainly decomposed by pyrolysis and depolymerization in the temperature ranges of 150 to 250 °C and above 450 °C, respectively.

The thermal stability of the NFs was investigated by TGA as shown in Fig. 5. The first weight loss of 6% at approximately 100 °C occurred due to the evaporation of absorbed water^35^. The following weight loss of 52% occurred in the temperature range of 200 to 350 °C, which involved the dehydration of PVA. The dehydrated PVA, *i*.*e*., polyene, was thermally decomposed by chain scission above 380 °C. The main decomposition of neat CNCs, associated with the pyrolysis of CNCs, occurred in the lower temperature range of 150 to 250 °C, and resulted from the catalytic effect of the sulfate groups of CNCs^36^. In the temperature range above 450 °C, CNCs were decomposed into carbon dioxide, thereby depolymerizing the cellulose chain^31,37^. Compared with neat PVA NFs, PVA/CNC NFs were decomposed at higher temperatures by approximately 285 and 485 °C for the second and third weight losses, respectively. These thermal behaviors of neat PVA and PVA/CNC composite NFs were in accordance with several previous studies which reported that the thermal stabilities of composites are improved by the hydrogen bonds between the matrices and fillers^12,38,39^. According to recent studies^29,31^, the thermal stability of a PVA/CNC composite is related to the dispersion homogeneity of CNCs in the composite. For instance, after CNC loading in a PVA/CNC composite exceeds an optimal CNC content of 12%, the thermal stability of the PVA/CNC composites decreases with increasing CNC loading due to separation of the CNC degradation peak, which involves the pyrolysis of CNCs, from the dehydration peak of the PVA/CNC composite^31^. The temperature of the separated degradation peak is lower than that of the main degradation peak of neat PVA. In the current study, the DTG curves of PVA/CNC NFs did not show any separation of the dehydration peaks, as can be seen in Fig. 5b, which indicates that the CNCs in the PVA/CNC NFs were homogeneously dispersed.

**Figure 6:**
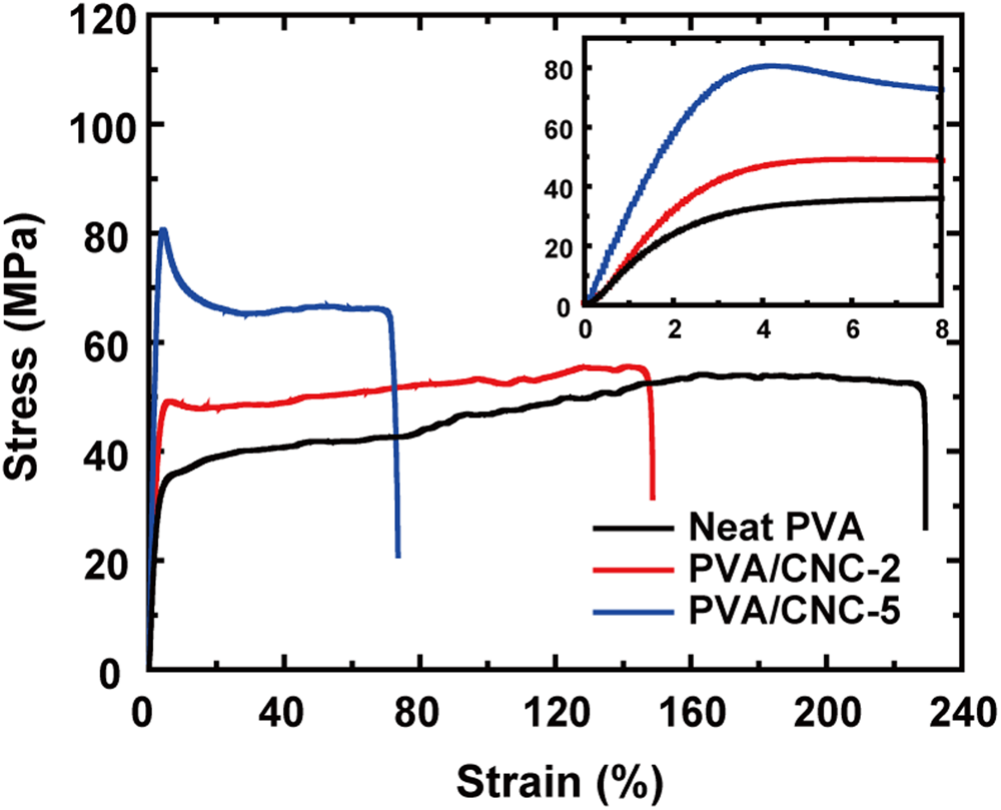
Stress-strain curves of neat PVA and PVA/CNC composite films. In the CNC content range of 0 to 5%, the Young’s modulus and tensile strength values increase with increasing CNC content. The inset shows an enlarged region of low strain where Young’s moduli are obtained.

A recent calculation predicted that polymers with more rigid backbones have higher *k* and thermal stability due to the confinement of molecular chains and longer phonon mean path^40^. Based on this calculation, our TGA results and the enhanced *k*, of which the details are discussed in the next paragraph, suggest that CNCs in the composites stiffen the flexible molecular chains of PVA through hydrogen bonds between CNCs and PVA. CNC is a significantly rigid material to the extent that the axial Young’s modulus of CNC ranges from 120 to 220 GPa, which is higher than that of Kevlar^41–44^. Additionally, hydrogen bonds between PVA and CNCs are possibly stronger because –OH groups in PVA molecular chains form hydrogen bonds with ring oxygen atoms in rigid anhydroglucose units in CNCs^32,33^. To further investigate the enhancement of mechanical properties, the stress-strain curves of neat PVA and PVA/CNC composite films were obtained as shown in Fig. 6. Relevant mechanical properties, taken from the stress-strain curves, are listed in Table 1. The Young’s modulus values of the PVA/CNC-2 and PVA/CNC-5 films are higher than that of the neat PVA film by 13% and 83%, respectively; the stiffness of the PVA/CNC composite films increased with increasing content of CNCs in the range of 0 to 5%. Considering that they are made of the same solutions, we assumed that NFs had mechanical properties similar to those of the films. Moreover, electrospun NFs may show enhancement of mechanical properties via addition of CNCs stronger than that of the films because CNCs can be dispersed more homogeneously during electrospinning^29^. More details regarding the measurement of the mechanical properties are provided in the Supplementary Information.

**Table 1:** Mechanical properties of neat PVA and PVA/CNC composite films.

Samples	Young’s modulus (GPa)	Tensile strength (MPa)	Elongation at break (%)
Neat PVA	1.69 ± 0.19	53.7 ± 1.81	173 ± 28
PVA/CNC-2	1.91 ± 0.13	56.8 ± 1.93	168 ± 22
PVA/CNC-5	3.10 ± 0.08	81.8 ± 2.06	92 ± 48

**Figure 7:**
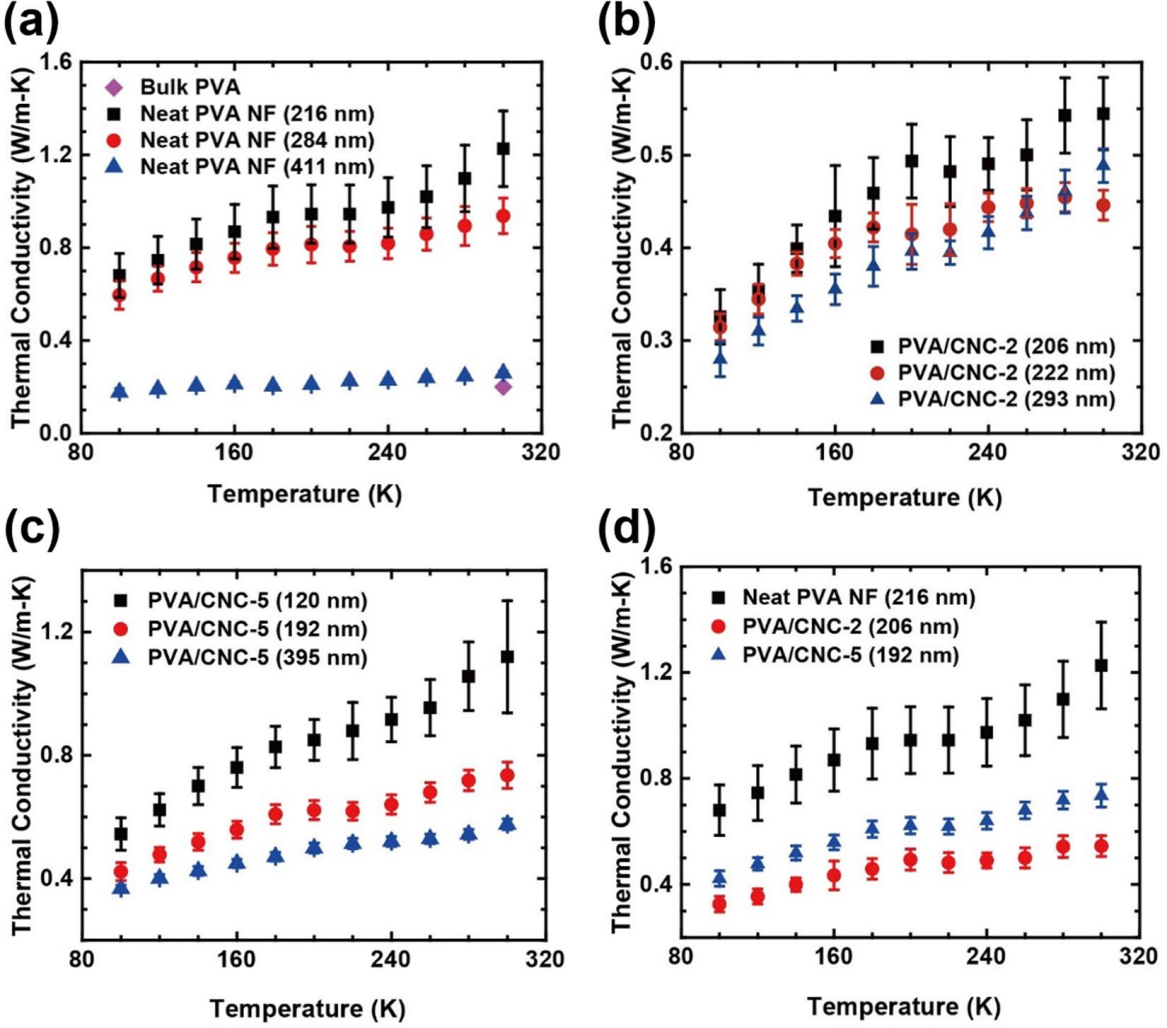
Thermal conductivity of (**a**) neat PVA NFs, (**b**) PVA/CNC-2 NFs, (**c**) PVA/CNC-5 NFs, and (**d**) neat PVA and PVA/CNC NFs with similar diameters of approximately 200 nm as a function of temperature. As shown in Fig. 7d, the thermal conductivity of the neat PVA NF with the smallest diameter showed the highest degree of *k* enhancement, which is due to the increased crystallinity. Although the degree of *k* enhancement was reduced in the PVA/CNC NFs, the *k* values of the PVA/CNC NFs were still higher than that of bulk PVA by factors of 2.5 and 3.5.

As shown in Fig. 7a, the *k* values of the neat PVA NFs increased with decreases in their diameter. The neat PVA NF with a diameter of 216 nm had a higher *k* of 1.23 W/m-K, which is almost six times higher than that of bulk PVA. While the *k* values monotonously increased with increasing temperature, it is expected that further reduction of diameter may yield a peak (originating from higher crystallinity and Umklapp scattering) in *k* as a function of temperature as was observed in previous reports^17,19^. For both the neat PVA and PVA/CNC composite NFs, thinner NFs also showed higher thermal conductivities as seen in Fig. 7b,c. The crystallinity of the neat PVA NFs increased with decreasing diameter, leading to an increase in *k*^45^. Similarly, the amorphous phase can be axially oriented by the confinement of diameter^46–48^, which may cause an increase in the thermal conductivity of the PVA/CNC NFs with decreasing diameter. The neat PVA NFs had the highest *k* among the NFs with similar diameters of approximately 200 nm as shown in Fig. 7d. The results suggest that the crystallinity of molecular chains has a greater positive influence on *k* than the addition of CNCs. As expected, PVA/CNC-5 NF had a slightly higher *k* than PVA/CNC-2 NF. In addition to the higher *k* of CNC in comparison to that of PVA^49^, this phenomenon is also explained by the content of CNC which satisfies the percolation threshold (*Φ*_c_). For rod-like nanoparticles, *Φ*_c_ is defined as *Φ*_c_ = 0.7/(*L*/*d*), where *L* and *d* are the length and the diameter of a nanoparticle, respectively^50^. With the geometries of the NFs, *Φ*_c_ is calculated to be in the range of 0.88% to 3.15%. The CNC content in PVA/CNC-2 NFs would not surpass the percolation threshold, resulting in a lower *k* than that of PVA/CNC-5 NFs.

**Figure 8:**
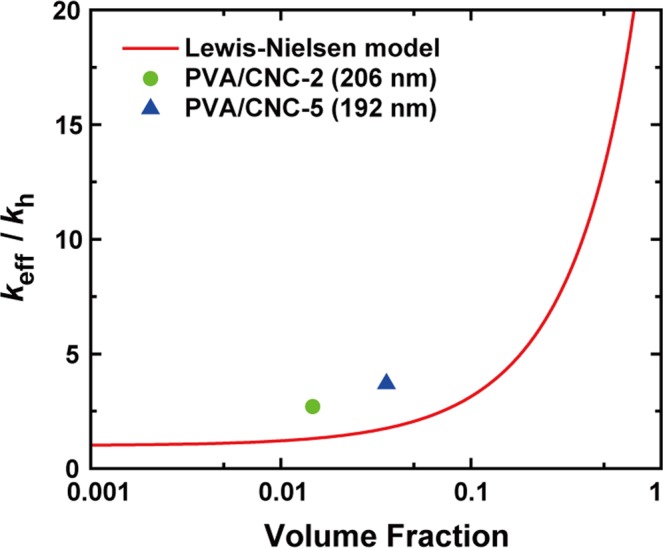
Thermal conductivity enhancement of PVA/CNC composite as a function of CNC volume fraction. The experimental results were higher than the Lewis–Nielsen model prediction approximately by a factor of 2.

To better understand the effect of the strong interaction between PVA and CNCs, the effective thermal conductivity (*k*_eff_) of the PVA/CNC composite was calculated based on the Lewis–Nielsen model^51^, which includes the shape and packing type of dispersed particles as fitting parameters. Here, *k*_eff_ is expressed as

1 $$\frac{{k}_{{\rm{eff}}}}{{k}_{{\rm{h}}}}=\frac{1+AB{\varphi }_{{\rm{f}}}}{1-B\psi {\varphi }_{{\rm{f}}}}$$

where *A* is the parameter related to the shape of the filler particles, and *B* is the parameter given by $B\equiv [({k}_{{\rm{f}}}/{k}_{{\rm{h}}})-1]/[({k}_{{\rm{f}}}/{k}_{{\rm{h}}})+A]$. Here, *k*_h_ and *k*_f_ are the *k* values of the host material and the filler, respectively, and *ψ* is defined as $\psi \equiv 1+[(1-{\varphi }_{{\rm{m}}})/{\varphi }_{{\rm{m}}}^{2}]{\varphi }_{{\rm{f}}}$, where *ϕ*_f_ and *ϕ*_m_ are the filler volume fraction and the maximum packing fraction, respectively. In the calculation, *k*_h_ and *k*_f_ were taken to be 0.2 and 5.7 W/m-K^14,49^, which are the *k* values of bulk PVA and an individual CNC, respectively. Additionally, *A*, *i*.*e*., $A\equiv 2L/d$, and *ϕ*_m_ were chosen to be 85.7 and 0.82 considering the orientation, aspect ratio, and packing type of CNCs^51^. As shown in Fig. 8, the Lewis–Nielsen model predicted that *k*_eff/_*k*_h_ had the upper limit value of 21.8, which corresponds to the effective thermal conductivity *k*_eff_ of 4.36 W/m-K, when the volume fraction became the maximum packing fraction of 0.82. Also, the prediction deviated significantly from the experimental results, which were approximately two times higher than the *k*_eff_ values predicted by the Lewis–Nielsen model. These high *k* values were obtained from the PVA/CNC composites with low volume fractions of CNCs in the proximity of the formation of a percolation network where the Lewis–Nielsen model is valid^52,53^. In this regard, it was thought the hydrogen-bond-assisted PVA/CNC networks would result in an increase in the backbone rigidity of the composites and correspondingly enhance their *k* values.

We measured the *k* values of individual neat PVA and PVA/CNC NFs using the suspended microdevice technique without any damage to the NF crystallinity and significant thermal contact resistances between the NFs and the suspended microdevices. The *k* values of the neat PVA NFs also were higher than those of bulk PVA and the PVA/CNC NFs due to their higher crystallinity. In contrast, the *k* enhancement of the PVA/CNC NFs is attributed to an increase in the backbone rigidity, which was enabled by the hydrogen bonds between PVA molecules and CNCs, considering that the PVA/CNC NFs have lower crystallinity than that of bulk PVA. Additionally, the axial orientation of the amorphous phases in the PVA/CNC NFs may increase the thermal conductivity as the diameters decrease. Therefore, both the thermal and mechanical properties of the PVA/CNC NFs can be improved simultaneously. Overall, this study demonstrated *k* enhancement in electrospun composite NFs for the first time and elucidated the mechanisms of the *k* increase.

## Methods

### CNC synthesis

CNCs were isolated from microcrystalline cellulose (MCC) using a hydrolysis method reported by Bondeson *et al*.^54^ In brief, 60 g of MCC was sufficiently soaked in 600 mL of DI water with mild stirring for 30 min. The MCC/water-suspension was transferred to a three-necked round flask and cooled in an ice bath for 30 min. Concentrated sulfuric acid (95–98%, 540 mL) was slowly added dropwise until the acid suspension reached the concentration of 9.2 M. The reaction flask was placed in an oil bath (40 °C), and the suspension was hydrolyzed for two hours at 40 °C under vigorous stirring. The suspension was cooled externally with an ice bath. Then, the suspension having crystalline residues was washed with DI water by washing/centrifugation cycles; the supernatant was removed from the sediment, replaced by new DI water, and mixed until a pH level of approximately 4.0 was achieved. The suspension (1% (w/v)) was processed through a high-pressure homogenizer with a diamond process nozzle D5 (130 micron) under pressure of 20,000 psi. The suspension passed through the interaction chambers and was also subject to homogenization at a rate of 400 mL min^−1^ at 10 °C for 8 passes to obtain CNCs. The product was then stored at 4 °C for further analysis and composite preparation. Using laser-Doppler-velocimetry (LDV), the ζ-potential of the CNCs in water was measured for the dispersion at 25 °C. For the calculation of the ζ-potential from the electrophoretic mobility, the dielectric constant and viscosity of water were obtained from the literature^55^. It was possible to ensure a stable dispersion state at the individual nanocrystal level because the CNCs had a sufficiently negative surface charge (ζ-potentials of −46.4 mV) for a clear homogeneous solution owing to abundant sulfate groups on the cellulose surface.

### Electrospinning process

A 5% (w/w) PVA (Sigma-Aldrich, *M*_w_ = 146,000–186,000, 99+% hydrolyzed) aqueous solution was used for the synthesis of neat PVA NFs. PVA/CNC NFs were made of solutions that were made by mixing 25 g of 5% (w/w) PVA aqueous solutions with 2.5 g and 6.25 g of 1% (w/w) CNC aqueous solutions, respectively. The corresponding weight ratios of CNC to PVA in the PVA/CNC aqueous solutions were 2% (w/w) and 5% (w/w), respectively. Using the solutions, NFs were electrospun (NanoNC Co., ESR100D) under the following conditions. The applied voltage was 22 kV, the syringe tip-collector distance was 20 cm, and the pumping speed was 3 μL/min. A higher voltage was chosen because, in a previous work^19^, a higher *k* was obtained from NFs that were electrospun under a high electrical voltage. While individual NFs were selected for *k* measurements, electrospun-NF mats with a thickness of ~50 μm were also prepared with the same electrospinning conditions for material characterizations.

### Characterizations

FTIR spectroscopy (Thermo Scientific, Nicolet iS10) was used with the attenuated total reflectance (ATR) mode to reveal the crystallinity of the NFs and to determine the characteristics of hydrogen bonds in the NFs. TGA analysis (Mettler Toledo, SDTA851e) was also performed at a heating rate of 10 °C/min under aerobic condition to investigate the intermolecular bonding, which affected the *k* of the NFs as well as their thermal stability. DSC analysis (TA Instrument Inc., Q20) was carried out with three heating–cooling cycles in the temperature range of 30 to 240 °C to investigate the melting and crystallization behavior of the samples (Fig. S5). All the samples were characterized with weights of 5 to 6 mg under a nitrogen purge gas flow of 50 mL/min. The ramping rate was set to 10 K/min. Temperature above 200 °C may cause the thermal degradation of PVA^56^, leading to distortion of measured properties. To minimize the adverse effect of thermal degradation, the maximum temperature of the cyclic DSC measurements was set to 240 °C. However, since the relatively low maximum temperature may yield the underestimation of crystallinity due to an incomplete melting process, non-cyclic DSC measurements were also performed in the temperature range of 30 to 300 °C for determining crystallinity as shown in Fig. 4a (excluding the range of thermal degradation above 250 °C). The degree of crystallinity (χ_c_ is given as^57^

$${\chi }_{c}( \% )=\frac{{\rm{\Delta }}{H}_{m}}{w{\rm{\Delta }}{H}_{m}^{0}}\times 100,$$

where ${\rm{\Delta }}{H}_{m}$ is the heat of fusion, *w* is the weight fraction of PVA in the PVA/CNC composite, and ${\rm{\Delta }}{H}_{m}^{0}$ is the heat of fusion for PVA with a crystallinity of 100%. The heat of fusion of PVA with a crystallinity of 100% $({\rm{\Delta }}{H}_{m}^{0})$ is 161 J/g, as in a previous study^58^. The morphologies of CNCs and NFs were observed using transmission electron microscopy (TEM; JEOL, JEM-3011 or JEM-2100F) operated at accelerating voltages of 200 kV and 100 kV, respectively. Also, the dispersion of CNCs in the NFs was examined with TEM. After embedding the electrospun PVA/CNC NFs in epoxy resin, a microtome equipped with a glass or gem grade diamond knife was used to cut a thin section, of which the thickness was typically 60 to 100 nm. This section was stained with 0.2% (w/w) uranyl acetate and deposited on a thin-carbon-coated 200 mesh copper grid. The sample was observed with TEM (Technai, G2T-20S) operated at an accelerating voltage of 100 kV. The image was taken under diffraction contrast in bright-field mode without prior contrast enhancement.

### Assembly of the NFs on suspended microdevices and *k* measurement

As suspended microdevices were attached to the collector of the electrospinning setup, electrospun NFs were laid on the suspended microdevices as shown in Fig. 1b. Using a micromanipulator, a NF was moved above the two suspended membranes of a suspended microdevice. Subsequently, the unnecessary parts of the NF were cut using an Ar^+^ ion laser, which was installed in a Raman spectroscopic system (Renishaw, inVia Raman microscope), with a power of approximately 10 mW and a wavelength of 514.5 nm. A focused ion or electron beam, which has been previously used to enhance the thermal contacts between nanomaterials and suspended microdevices^59,60^, was not employed due to its annealing effect, which would result in the degradation of the *k* value of electrospun polymer NFs^19^. However, Zeng *et al*. recently reported that a thermal contact resistance accounted for 30% to 50% of the total resistance of an electrospun epoxy resin NF, which was assembled on a suspended microdevice without the aid of electron-beam-assisted metal deposition^23^. Therefore, cyclohexane, which is a non-polar solvent, was dropped on the NFs and the suspended microdevices to reduce the contact thermal resistances between the NFs and the substrates. While the cyclohexane dried, the NFs adhered to the suspended microdevices due to capillary-force-induced van der Waals interactions. The use of a polar solvent, such as ethanol or methanol, was avoided because it would change the morphology of electrospun NFs^61^. Due to the low *k* and nanometer-scale diameter, the thermal resistance of the polymer NFs was high enough to neglect the contact thermal resistances (Supplementary Information). Due to the low thermal conductance of NFs, it was necessary to eliminate the contribution of background thermal conductance from the total thermal conductance. The details of the background thermal conductance measurement and elimination are presented in the Supplementary Information.

### Stress and strain curve measurement

Tensile property measurements were conducted with a universal testing machine (Withlab Co., WL2100) at 50% relative humidity and room temperature, in accordance with the ASTM D882, which is a standard method for measuring the tensile properties of thin films. All specimens were prepared with thicknesses of 0.08–0.12 mm. The test conditions were set to a strain rate of 25 mm/min, grip-to-grip distance of 50 mm, and load cell of 200 N.

## Supplementary information

Supplementary Information
