# Supplementary material for: Thermal conductivity enhancement in electrospun poly(vinyl alcohol) and poly(vinyl alcohol)/cellulose nanocrystal composite nanofibers
Source: Sci Rep. 2019 Feb 28;9:3026. doi: 10.1038/s41598-019-39825-8 (PMC6395720; doi:10.1038/s41598-019-39825-8)
Supplement: Supplementary file 1 — Supplementary Information [file 41598_2019_39825_MOESM1_ESM.docx]

**Supplementary Information**

**Thermal conductivity enhancement in electrospun poly(vinyl alcohol) and poly(vinyl alcohol)/cellulose nanocrystal composite nanofibers**

Yeongcheol Park,^1,†^ Myungil You,^1,†,‡^ Jihoon Shin,^2,3^ Sumin Ha,^4^ Dukeun Kim,^5^ Min Haeng Heo,^2,3^ Junghyo Nah,^6^ Yoong Ahm Kim,^4,*^ and Jae Hun Seol^1,*^

^1^School of Mechanical Engineering, Gwangju Institute of Science and Technology (GIST), Buk-gu, Gwangju 61005, Korea.

^2^Center for Environment & Sustainable Resources, Korea Research Institute of Chemical Technology, 141 Gajeong-ro, Yuseong-gu, Daejeon 34114, Korea.

^3^Department of Advanced Materials & Chemical Engineering, University of Science & Technology, 217, Gajeong-ro, Yuseong-gu, Daejeon 34113, Korea.

^4^Department of Polymer Engineering, Graduate School, Chonnam National University, 77, Yongbong-ro, Buk-gu, Gwangju 61186, Korea.

^5^Smart Textile R&D team, Korea High Tech Textile Research Institute, 170, Geomjun-gil, Nam-myeon, Yangju-si, Gyeonggi-do 11410, Korea.

^6^Department of Electrical Engineering, Chungnam University, Yuseong-gu, Daejeon 34134, Korea

*Correspondence and requests for materials should be addressed to Jae Hun Seol (email: [jhseol@gist.ac.kr](mailto:jhseol@gist.ac.kr)) and Yoong Ahm Kim (email: [yak@chonnam.ac.kr](mailto:yak@chonnam.ac.kr))

^†^These authors contributed equally to this work.

^‡^Present address: Electric Power Conversion System Engineering Design Team, Hyundai Motor Group, 150, Hyundaiyeonguso-ro, Namyang-eup, Hwaseong-si, Gyeonggi-do, 18280, Korea.

**List of prepared samples**

| Sample | Filler, % (w/w) | Length/Diameter (μm/nm) |
| --- | --- | --- |
| Neat PVA-1 | None | 3.3/216 |
| Neat PVA-2 | None | 5.9/284 |
| Neat PVA-3 | None | 3.3/411 |
| PVA/CNC 2%-1 | CNC, 2% (w/w) | 6.9/206 |
| PVA/CNC 2%-2 | CNC, 2% (w/w) | 4.1/222 |
| PVA/CNC 2%-3 | CNC, 2% (w/w) | 9.0/293 |
| PVA/CNC 5%-1 | CNC, 5% (w/w) | 3.9/120 |
| PVA/CNC 5%-2 | CNC, 5% (w/w) | 5.2/192 |
| PVA/CNC 5%-3 | CNC, 5% (w/w) | 6.7/395 |

Table S1. Filler contents and geometries of electrospun NFs.

**TEM characterization of CNCs and NFs**

To take TEM images of CNCs, a drop of 0.01% (w/w) CNC dispersion was deposited on a thin-carbon-coated 200 mesh copper grid, which was negatively stained with 0.2% (w/w) uranyl acetate and allowed to dry. The image shown in Fig. 2a (Manuscript) was taken under diffraction contrast in the bright-field mode without prior contrast enhancement. To homogeneously disperse the CNCs in water to a level of approximately 0.1% (w/w), the dispersion was evaporated to a 0.2% (w/w) consistency. As shown Fig. S1a,b, TEM images of electrospun nanofibers (NFs) were taken as well. However, the dispersion of CNCs in the NFs cannot be observed in the TEM images. Therefore, microtoming and subsequent dyeing with uranyl acetate was applied as described in the Manuscript. Thus, CNCs in the PVA/CNC-5 NFs became discernable as shown in Fig. S1c.


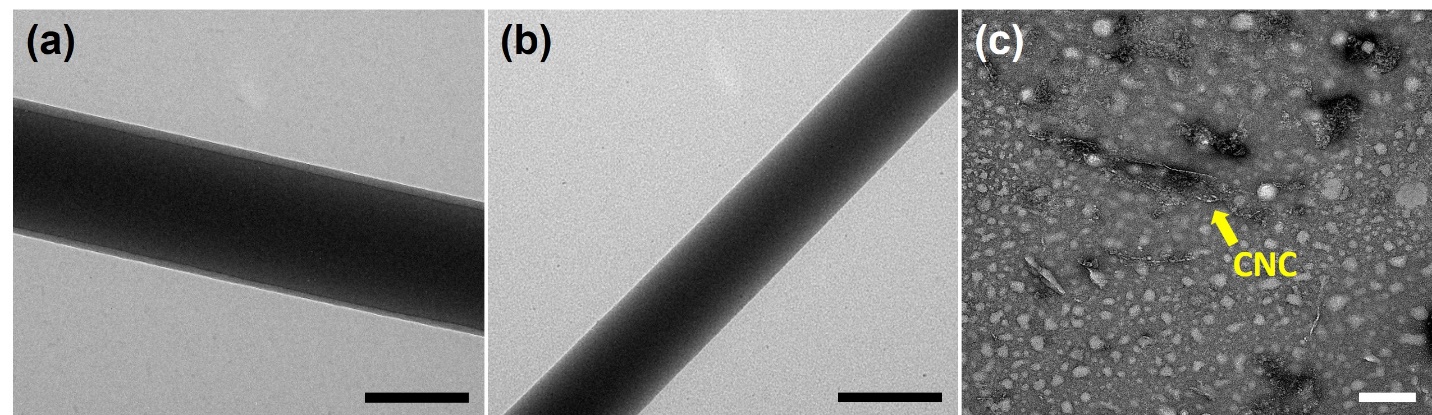


**Figure S1.** TEM images of an electrospun PVA/CNC-2 NF (**a**), PVA/CNC-5 NF (**b**), and the dispersion of PVA/CNC-5 NF (**c**). The contrast in brightness in (a) would be due to a core-shell structure caused by the change in CNC density. The scale bars in (a–c) are 200 nm.

**Effect of electron beam irradiation on the thermal conductivity of an electrospun nanofiber**

Generally, an electron beam or ion beam is used to make metal- or carbon-deposited electrical and thermal contacts between a nanomaterial and the electrodes of a suspended microdevice^1,2^. However, Ma *et al*. reported that the thermal conductivity (*k*) of an electrospun polyethylene NF was significantly reduced by exposure to an electron beam^3^. The aligned molecular chains in the electrospun NF were annealed by an electron beam, resulting in the degradation of crystallinity. In this study, we compared the *k* values of an electrospun poly(vinyl alcohol) (PVA) NF before and after exposure to an electron beam of 5 kV acceleration via scanning electron microscopy (SEM). Similar to the observation of Ma *et al.*, the *k* value of the PVA NF was diminished in comparison to that of bulk PVA after irradiation of the NF by an electron beam as shown in Fig. S2. Therefore, all the samples that were presented in the Manuscript were prepared without the assistance of an electron beam.


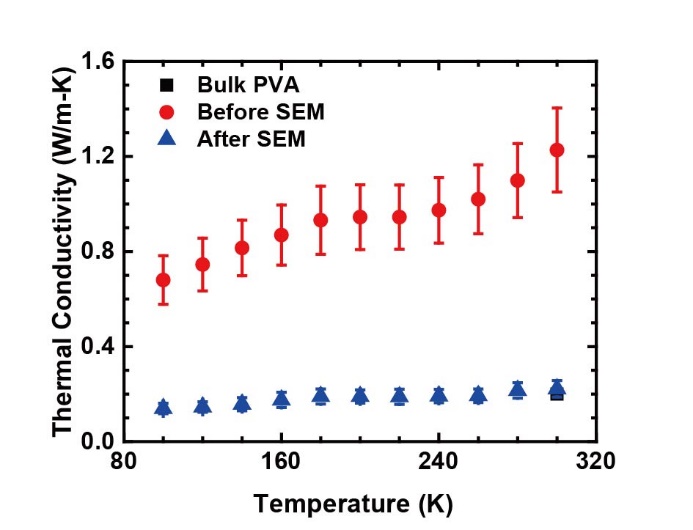


Figure S2. Thermal conductivity change in a neat PVA NF before and after electron beam irradiation. After exposure to an electron beam, the thermal conductivity of the NF was almost reduced to that of bulk PVA^4^.

**Estimation of thermal contact resistance**

An electron beam was not used due to concerns about crystallinity damage to the NFs as mentioned above. Although an NF would adhere to a substrate when a drop of cyclohexane dried, the contact thermal resistance would be higher than that obtained by electron-beam-assisted metal deposition. Thus, it was necessary to estimate the portions of contact resistance in the total thermal resistance that could be accounted for by contributions of the sample and contact. Depending on the extent of the contact resistance contribution, the contact resistance could be neglected. The contact resistance between a PVA NF and the silicon nitride substrate of a suspended microdevice was calculated as

$R_{\mathrm{contact}}=\frac{1}{\pi lk_{p}}\ln\left( \frac{4d}{w} \right)-\frac{1}{2lk_{p}}+\frac{1}{\pi lk_{s}}\ln\left( \frac{2d}{\pi w} \right)$, (S1)

where *k*_p_is the transverse thermal conductivity of the PVA NF, *k_s_* is the thermal conductivity of the silicon nitride (SiN_x_) substrate, *l* is the partial length of the NF in contact with the substrate, *d* is the diameter of the NF, and *w* is the contact width of the NF on the substrate^5^. As for the *k* values in Eq. S1, we assumed that *k*_p_ has the same *k* as that of bulk PVA, which is 0.2 W/m-K at room temperature, and the thermal conductivity of silicon nitride was assumed to be 4 W/m-K^6,7^. Regarding the geometries of a NF lying on a membrane, *l* and *d* were assumed to be 10 μm and 400 nm, respectively, and *w* is expressed as

$w= \left( \frac{16NE_{m}d}{\pi l} \right)^{1/2}$, (S2)

where *N* is the normal load, and *E*_m_ is the effective modulus based on an elastic plane strain analysis. Here, *N* primarily originates from the van der Waals interaction (*F*_vdW_) between the NF and SiN_x_, *i.e.*, *N*$\approx$*F*_vdW_, because it is dominant over the body forces, such as gravitational forces, in the micron length scale^8,9^. Furthermore, *F*_vdW_ and *E*_m_ are given as

$F_{\mathrm{vdW}}=\frac{A_{p-s}ld^{1/2}}{16h^{5/2}}$, (S3)

$E_{m}=\frac{1}{2}\left( \frac{1 - \upsilon_{s}^{2}}{E_{s}}+\frac{1 - \upsilon_{p}^{2}}{E_{p}} \right)$, (S4)

where *A*_p-s_ is the Hamaker constant for the heterogeneous van der Waals interaction between different materials, and *h* is the gap size^10,11^. In the above equations, *v*_p_ and *v*_s_*,* are the Poisson’s ratios of PVA and SiN_x_, respectively, and *E*_p_ and *E*_s_ are the Young’s moduli of the PVA NF and the SiN_x_ substrate, respectively. In Eq. S3, *h* is approximated to the sum of the van der Waals radii of a hydrogen atom of PVA and a nitrogen atom of SiN_x_, and *A*_p-s_ is estimated as the geometric mean approximation of $A_{p-s}\approx\sqrt{A_{p-p}A_{s-s}}$ ^9,10,12^, where *A*_p-p_ and *A*_s-s_ are the Hamaker constants for homogeneous interaction of PVA and SiN_x_, respectively. We used the Hamaker constant for polyvinyl chloride-polyvinyl chloride instead of PVA-PVA due to the absence of information^10^. Also, *E*_s_ and *v*_s_ were 255 GPa and 0.23, respectively, which were taken from those of low stress SiN_x_^13^. Here, *E*_p_ was chosen to be about 3 GPa considering the size effect that the Young’s modulus of an NF tends to increase as the diameter decreases^14^, and *v*_p_ was set to be 0.45^15^. Through Eqs. of S2–4, *w* was estimated to be approximately 32 nm. Consequently, the calculated total contact resistance (2*R*_contact_) was about 7.8$\times$10^5^ K/W. As the thermal resistances of the samples, which are the reciprocals of measured thermal conductance (*G*) values, were in the range from 7.3$\times$10^7^ to 3.8$\times$10^8^ K/W, the calculated contact resistance accounted for approximately 0.2% to 1.1% of the total thermal resistance. Therefore, it was justified to neglect the thermal contact resistances of the NFs, and this negligence resulted in a *k* underestimation of only 0.2% to 1.1%.

**Negligible effect of cyclohexane on the morphology of PVA nanofibers**


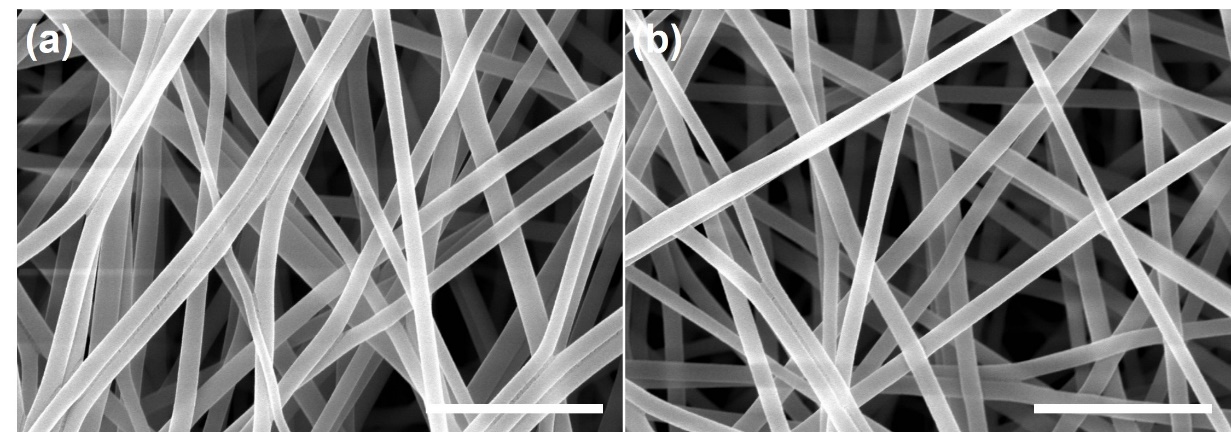


**Figure S3.** SEM images of (**a**) pristine electrospun PVA NFs and (**b**) electrospun PVA NFs which were soaked in cyclohexane and dried afterward. The morphology of the two different NFs was not changed. The scale bars of both images are 2 μm.

**Difficulty in the deconvolution of Fourier transform infrared (FTIR) spectra for determining the crystallinity of PVA/CNC NFs**


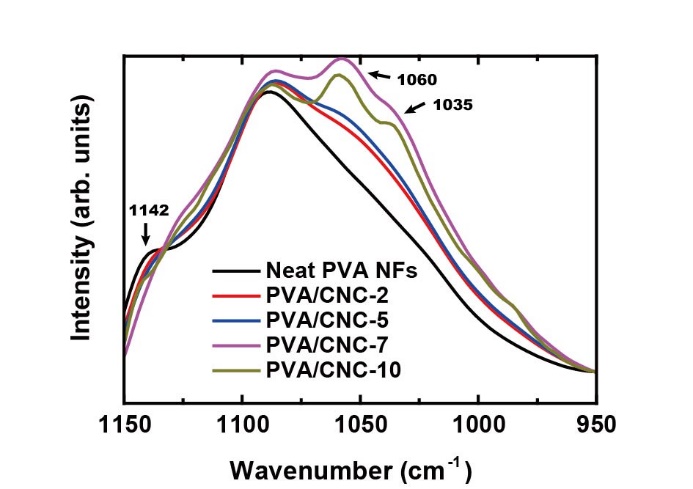


**Figure S4.** FTIR spectra of electrospun PVA NFs and PVA/CNC NFs in the range of 950 to 1150 cm^-1^. As the CNC content increases above 7%, the protrusion of peaks at 1060 and 1035 cm^-1^, which originate from the secondary and primary –OH groups of CNCs, respectively, hinders the deconvolution of the peaks at 1142 and 1094 cm^-1^. The intensity ratios of the peaks (the peak intensities at 1142 cm^-1^ to those at 1094 cm^-1^) are used to determine crystallinity.

**Multi-cyclic differential scanning calorimetry (DSC) measurement**

To investigate the crystallization of PVA/CNC NFs, DSC measurement was attempted with three heating–cooling cycles as shown in Fig. S5 and Table S2. The cyclic DSC measurements were performed with a ramping rate of 10 K/min under a nitrogen purge gas flow of 50 mL/min, which was also applied to the non-cyclic DSC measurements (Manuscript). Since PVA is susceptible to degradation at temperatures above 200 °C^16^, the DSC measurements were conducted for three cycles in the temperature range of 30 to 240 °C without the isothermal step at 240 °C. The first heating cycle for all samples showed a small and wide peak caused by the evaporation of bound water. In comparison with bulk PVA, the neat PVA NFs had higher crystallinity, *i.e.*, the higher heat of fusion in DSC measurements, which was due to the crystallinity increase induced by chain alignment during electrospinning^17^. The higher crystallization temperature (*T*_c_) and sharper crystallization peaks also evidenced the higher crystallinity of the electrospun neat PVA NFs. The addition of CNCs reduced the crystallinity of the electrospun PVA NFs to a degree lower than that of bulk PVA because the CNCs disturbed the chain alignment during electrospinning^18–20^. As the number of cycle increased, both enthalpies and peak temperatures, which are related to melting and crystallization, decreased due to the thermal degradation of PVA^16^. However, for the PVA/CNC NFs, the crystallinity slightly increased after the first heating–cooling cycle, which was attributed to the role of CNCs as nucleation sites for the crystallization of PVA^21^.


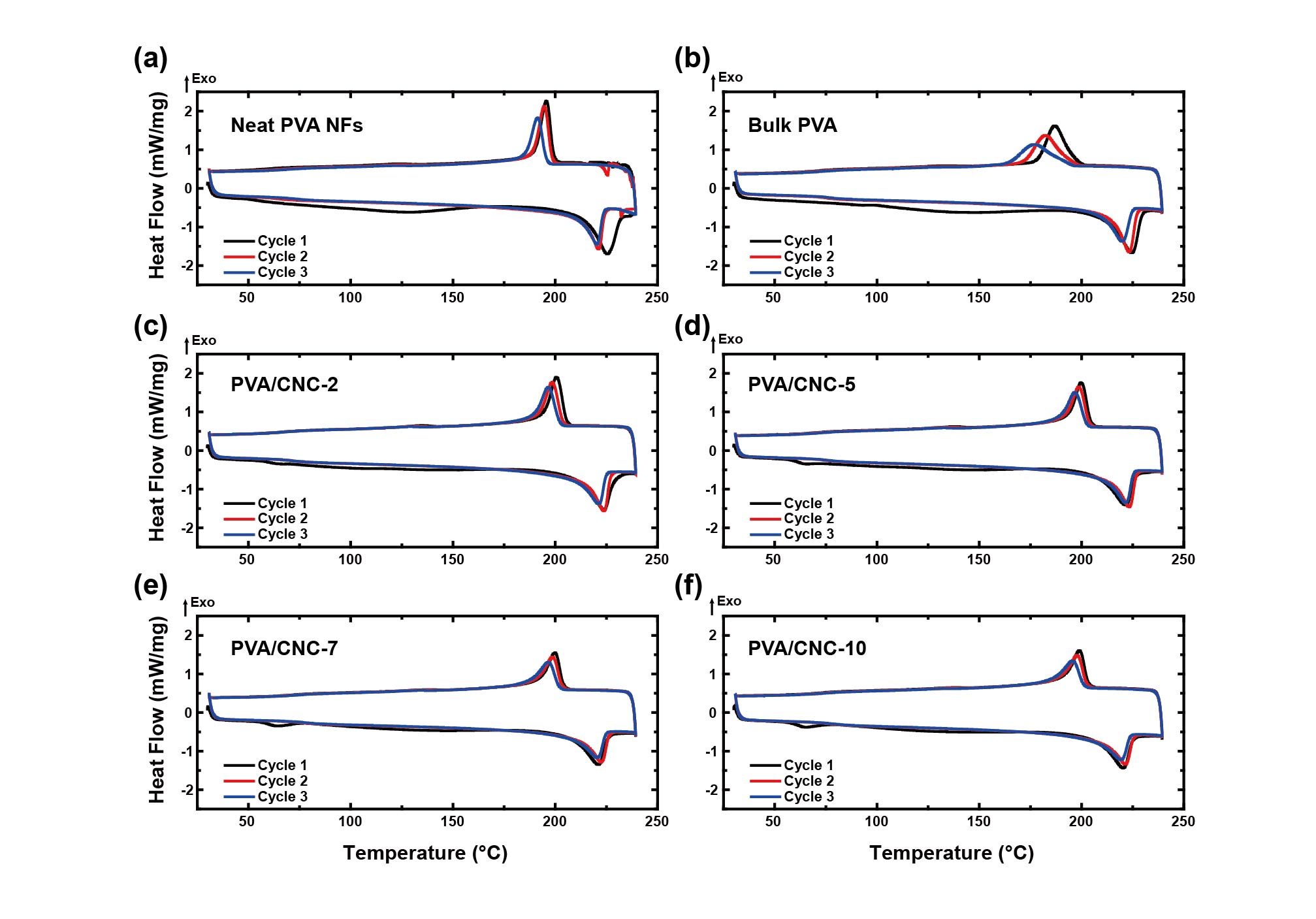


**Figure S5.** Differential scanning calorimetry (DSC) curves of neat PVA NFs, bulk PVA, and PVA/CNC NFs through three heating–cooling cycles in the temperature range of 30 to 240 °C.

| **Sample** | **Cycle** | $\boldsymbol{T}_{\mathbf{m}}$ **(°C)** | $\boldsymbol{\Delta}\boldsymbol{H}_{\mathbf{m}}$ **(J/g)** | $\boldsymbol{T}_{\mathbf{c}}$ **(°C)** | $\boldsymbol{\Delta}\boldsymbol{H}_{\mathbf{c}}$ **(J/g)** |
| --- | --- | --- | --- | --- | --- |
| **Neat PVA NFs** | **1^st^** | 225.34 | 82.899 | 195.62 | 69.956 |
|  | **2^nd^** | 221.16 | 67.644 | 194.60 | 68.463 |
|  | **3^rd^** | 220.39 | 59.165 | 191.34 | 62.991 |
| **Bulk PVA** | **1^st^** | 224.54 | 80.892 | 186.86 | 77.013 |
|  | **2^nd^** | 223.21 | 61.107 | 182.14 | 70.510 |
|  | **3^rd^** | 219.93 | 55.895 | 176.52 | 62.084 |
| **PVA/CNC-2** | **1^st^** | 223.89 | 66.400 | 200.59 | 74.291 |
|  | **2^nd^** | 223.75 | 68.358 | 198.52 | 68.230 |
|  | **3^rd^** | 221.26 | 64.623 | 196.47 | 66.086 |
| **PVA/CNC-5** | **1^st^** | 221.60 | 60.964 | 199.76 | 68.615 |
|  | **2^nd^** | 223.29 | 62.386 | 198.57 | 68.342 |
|  | **3^rd^** | 221.61 | 59.908 | 196.63 | 64.846 |
| **PVA/CNC-7** | **1^st^** | 221.05 | 54.296 | 199.81 | 63.818 |
|  | **2^nd^** | 222.53 | 56.081 | 198.61 | 62.804 |
|  | **3^rd^** | 220.62 | 53.353 | 196.52 | 59.297 |
| **PVA/CNC-10** | **1^st^** | 220.42 | 54.563 | 198.87 | 68.354 |
|  | **2^nd^** | 221.41 | 56.274 | 197.74 | 66.809 |
|  | **3^rd^** | 219.37 | 54.008 | 195.45 | 61.729 |

**Table S2.** Cyclic DSC measurement results for neat PVA NFs, bulk PVA, and PVA/CNC NFs.

**Fourier transform infrared (FTIR) study on hydrogen bonds between PVA and CNCs.**


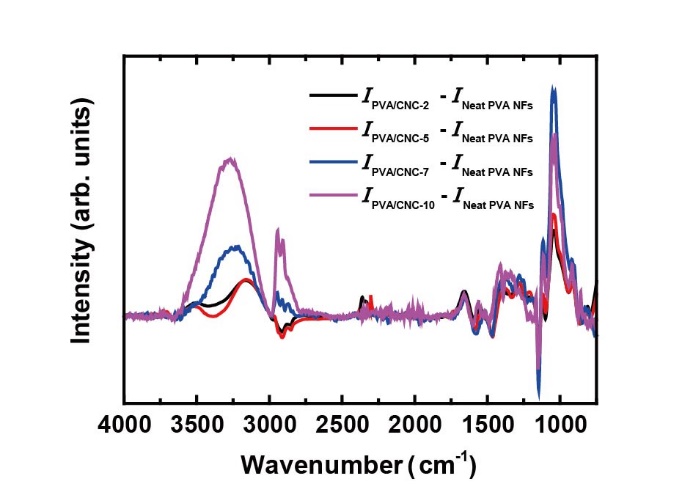


Figure S6. FTIR spectra of the PVA spectrum subtracted from the PVA/CNC NFs spectra. The range of 3000 to 3600 cm^-1^ provides information on the formation of inter- and intramolecular hydrogen bonds between PVA and CNCs.

Peresin *et al*. subtracted the FTIR spectrum of PVA/CNC-15% (w/w) composite NFs from that of neat PVA NFs, which were respectively normalized with the peaks at 850 cm^-1^ and considered the difference between the two spectra of O–H band (3000–3600 cm^-1^) as the influence of hydrogen bonds^18^. As shown in Fig. S6, when the CNC content was below 5%, a discernable difference occurred in the range of 3000 to 3300 cm^-1^, which is characterized by the formation of inter- and intramolecular hydrogen bonds between CNCs and PVA molecules. As the CNC content increased above 7%, the prominent rise of the subtracted relative intensity was observed due to plentiful –OH groups of CNCs, indicating the existence of hydrogen-bonded –OH groups as well as free –OH groups.

Additionally, the formation of hydrogen bonds between PVA and CNCs was investigated through examination of the FTIR spectra associated with the C–O band (Fig. S7). According to a previous study^22^, the variation of the C–O band with respect to the CNC content in PVA/CNC composite NFs indicates the formation of hydrogen bonds between the –OH groups of PVA and oxygen of CNCs. However, we did not observe any meaningful peak shift of the C–O bands. This insensitivity of the peak position of the C–O band to changes in the CNC content may be ascribed to the coexistence of inter- and intramolecular hydrogen bonds and the relatively lower CNC content in comparison with that of the previous study^22^.


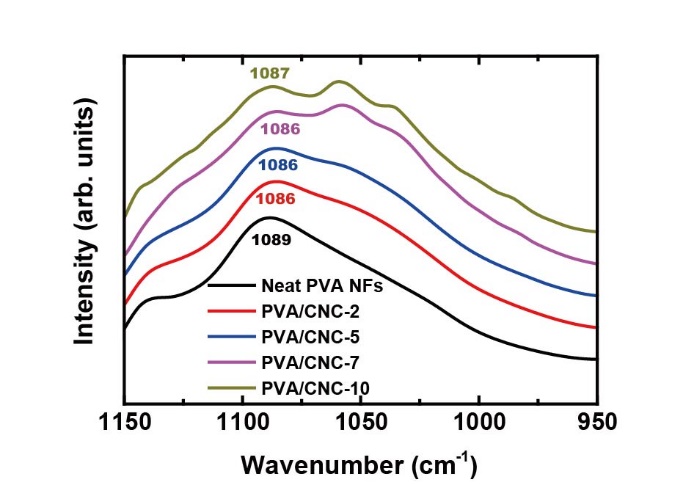


**Figure S7.** FTIR spectra of electrospun neat PVA and PVA/CNC NFs in the range of 950 to 1150 cm^-1^, which is associated with the C–O absorption band^22^. The CNC weight fractions in the current samples may not be sufficient to produce significant changes in the spectra.

**Measurement method**

In this study, suspended microdevices were used to measure the *k* values of electrospun NFs. Due to their low *k* values and small diameters, the NFs had *G* values as low as a few nW/K. There are two critical issues in the *G* measurement of such nanomaterials: measurement sensitivity and background heat transfer. Without geometric modifications, a standard suspended microdevice provides a sensitivity of ~ 1 nW/K ^1^, which is insufficient for measuring the *G* values of electrospun NFs as low as a few nW/K at room temperature. Therefore, the differential bridge circuit technique was applied to improve the measurement sensitivity^23^. As shown in Fig. S8, two platinum resistance thermometers (PRTs) on two suspended microdevices were located in a cryostat. The resistances of the PRTs on the heating and sensing sides of the suspended microdevice with the NF are denoted as *R*_h_ and *R*_s_, respectively; those of the other suspended microdevice, which did not have a NF, are denoted as *R*_h,ref_ and *R*_s,ref_, respectively. An NF bridged the heating and sensing side membranes of one of the two suspended microdevices, while the other had none. There was one 5-kΩ precision resistance (*R*_1_) and a potentiometer (*R*_2_), which varied in the range of 5 to 5.3 kΩ, outside the cryostat. We assumed that *R*_1_ and *R*_2_ were constant during the measurements. The bridge gate voltage (*v*_g_) was measured and applied to the relation between the resistances in the Wheatstone bridge circuit^23^. In this differential bridge circuit configuration, the measurement sensitivity of the sensing PRT was reduced by approximately a factor of 100 compared with that of the 4-point method. Despite the improved measurement sensitivity, there still was a background *G*, that is, parasitic heat transfer, which originated from residual gas conduction, thermal radiation, and the underlying substrate between the two suspended membranes. Therefore, the background *G* values were removed by measuring the sample *G* values with and without the Joule heating of *R*_s,ref_ as shown in Fig. S8. In addition to the differential bridge technique, which was introduced by Wingert *et al*.^23^, *R*_s,ref_ in the cryostat was simultaneously heated via the background *G* when the same ramping DC current as that applied to *R*_h_ flowed through *R*_h,ref_. In the aspect of heating reference resistances through background *G*s, this technique is similar to that reported by Weathers *et al*.^24^ However, this technique must separately measure *v*_g_ values with and without the reference heating, which are *v*_g,1_ and *v*_g,2_, respectively:

$v_{g,1}= \left( \frac{R_{s,ref} \left( 1+\alpha\right)}{{R_{s} \left( 1+\alpha+\beta\right) + R}_{s,ref} \left( 1+\alpha\right)}-\frac{R_{2}}{{R_{1} + R}_{2}} \right)v_{AC,s}$, (S5)

$v_{g,2}= \left( \frac{R_{s,ref}}{{R_{s} \left( 1+\alpha+\beta\right) + R}_{s,ref}}-\frac{R_{2}}{{R_{1} + R}_{2}} \right)v_{AC,s}$, (S6)

where *α* and *β* are the resistance increases due to DC heating through the background and sample *G*s, respectively, and $v_{AC,s}$ is the circuit-driving source voltage of 0.008 V. After the *α* and *β* values were obtained from the two measurements, the resistance increases were converted to corresponding temperature changes through the procedure reported by Wingert *et al.*^23^ Recently, an analogues technique was employed for the *k* measurement of an electrospun epoxy resin NF. In the study, the authors also applied a differential bridge circuit with heating to the on-chip pair resistance through the background *G*^25^. The Wheatstone bridge circuit configuration of their study was different from that adopted in this work, and their samples were measured once with heating applied to the on-chip pairs.


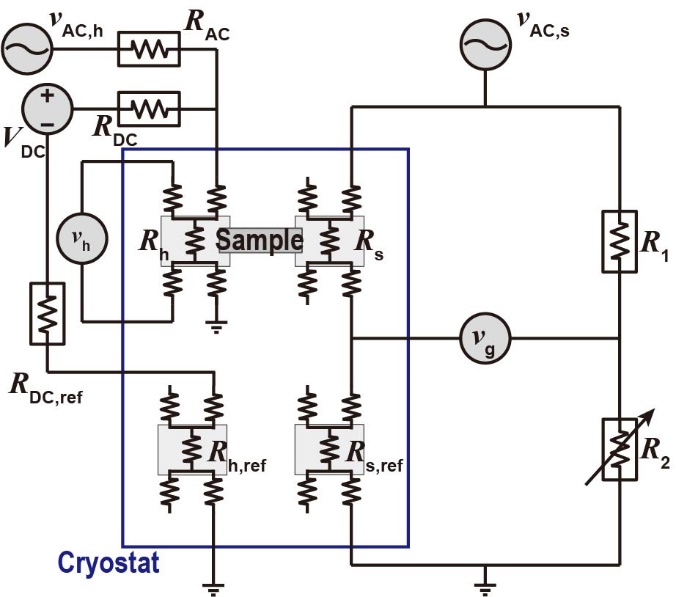


Figure S8. Schematic of the measurement setup, where *v*_g_ values were measured with and without reference heating, respectively. In this setup, *v*_AC,h_ $\boldsymbol{\approx}$ 5 V, *v*_AC,S_ $\boldsymbol{\approx}$ 0.008 V, *R*_AC_ $\boldsymbol{\approx}$ 10 MΩ, *R*_DC_ $\boldsymbol{\approx}$ *R*_DC,ref_ $\boldsymbol{\approx}$ 500 kΩ, and *R*_1_ $\boldsymbol{\approx}$ 5 kΩ. Before the measurement, *v*_g_ was adjusted to a few μV with *R*_2_.

**Uncertainty analysis regarding background conductance measurement**

According to previous reports^23,24^, the background *G* values of suspended microdevices were measured to be in the wide range of 0.2 to 0.8 nW/K, and they were affected by many factors, such as device geometry, vacuum level, radiation shielding, and so forth. Therefore, the background *G* in this study was measured, and it was determined to be 0.34±0.09 nW/K at 300 K, as shown in Fig. S9. As shown in the inset of Fig. S9, the background *G* has comparatively large random uncertainty due to its low signal-to-noise ratio. After the background *G* was measured, the sample *G* values were obtained by subtracting the background *G* from the total *G* values. It was assumed that all suspended microdevices have the same background *G* under the following conditions: they have the same geometries, they were made from one fabrication batch, and the measurement conditions were consistent. Accordingly, the uncertainty in each sample *G* was calculated as

$U_{G_{\mathrm{sample}}}=\sqrt{{U_{G_{\mathrm{bkg}}}}^{2}+{U_{G_{\mathrm{total}}}}^{2}}$ , (S7)

where $U_{G_{\mathrm{sample}}}$, $U_{G_{\mathrm{bkg}}}$, and $U_{G_{\mathrm{total}}}$ are the uncertainties of the sample, background, and total *G* values, respectively. For the uncertainty of *k* values, the geometric uncertainties were taken into account as well as the *G* uncertainties, which is expressed as

$\frac{U_{k}}{k}=\sqrt{\left( \frac{U_{G_{\mathrm{sample}}}}{G_{\mathrm{sample}}} \right)^{2}+\left( \frac{U_{L}}{L} \right)^{2}+\left( 2\frac{U_{d}}{d} \right)^{2}}$ , (S8)

where *L* is the length of the suspended part of the NF, and *U_k_*, *U_L_*, and *U_d_* are the uncertainties of the NF *k*, *L*, and *d*, respectively.


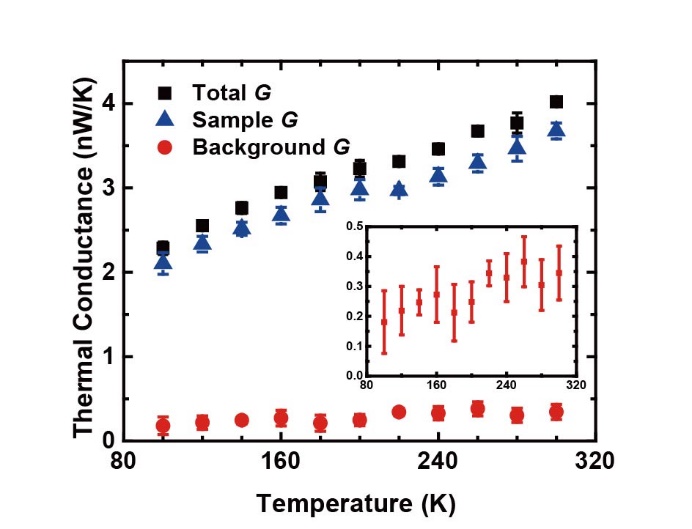


Figure S9. Total, sample, and background *G* values of PVA/CNC 2% NF with a diameter of 293 nm as a function of temperature. The sample *G* was obtained by subtraction of the background *G* from the total *G*. Inset shows the background *G* only. The horizontal and vertical axes of the inset represent temperature (K) and thermal conductance (nW/K), respectively.

**Stress-strain curves of neat PVA and PVA/CNC composite NFs**

As explained in the Manuscript, the *k* enhancement of PVA/CNC composite NFs is likely to be correlated to its mechanical property. According to previous PVA/CNC composites studies^19,26,27^, the mechanical properties of polymer composites highly depend on the filler contents as the measured *k* values in the current study. Therefore, it is necessary to investigate the mechanical properties of the samples. However, the mechanical property measurement of electrospun NFs requires a tensile testing equipment with an exceptionally high resolution due to the nanometer scale dimeters of the NFs. Considering the objective and scope of this study, it is sufficient to measure the tensile properties of PVA/CNC films, which were made of the same solutions as the NF samples, rather than measuring those of NFs. The measured mechanical properties of PVA/CNC composite films, which are listed in Table 1 (Manuscript), were obtained by averaging five measurement results for each sample. The results show that Young’s modulus and tensile strength increased with increasing CNC contents. Such phenomena are attributed to the formation of hydrogen bonded-networks between PVA and CNCs, which enables the effective stress transfer. Moreover, a decrease in the elongation at break with increasing CNC contents indicates that the PVA/CNC composite films became stiffer owing to an increase in the hydrogen bonds between PVA and CNCs.

**References**

1. Shi, L. *et al.* Measuring thermal and thermoelectric properties of one-dimensional nanostructures using a microfabricated device. *J. Heat Transf.* **125,** 881–888 (2003).

2. Mavrokefalos, A., Pettes, M. T., Zhou, F. & Shi, L. Four-probe measurements of the in-plane thermoelectric properties of nanofilms. *Rev. Sci. Instrum.* **78,** 034901 (2007).

3. Ma, J. *et al.* Thermal conductivity of electrospun polyethylene nanofibers. *Nanoscale* **7,** 16899–16908 (2015).

4. Mark, J. E. *Physical properties of polymers handbook*. **1076,** (Springer, 2007).

5. McGee, G. R., Schankula, M. H. & Yovanovich, M. M. Thermal resistance of cylinder-flat contacts: Theoretical analysis and experimental verification of a line-contact model. *Nucl. Eng. Des.* **86,** 369–381 (1985).

6. Mastrangelo, C. H., Tai, Y.-C. & Muller, R. S. Thermophysical properties of low-residual stress, silicon-rich, LPCVD silicon nitride films. *Sensors Actuators A Phys.* **23,** 856–860 (1990).

7. Sultan, R., Avery, A. D., Stiehl, G. & Zink, B. L. Thermal conductivity of micromachined low-stress silicon-nitride beams from 77 to 325 K. *J. Appl. Phys.* **105,** (2009).

8. Bahadur, V., Xu, J., Liu, Y. & Fisher, T. S. Thermal Resistance of Nanowire-Plane Interfaces. *J. Heat Transfer* **127,** 664 (2005).

9. Visser, J. Van der Waals and other cohesive forces affecting powder fluidization. *Powder Technol.* **58,** 1–10 (1989).

10. Israelachvili, J. N. *Intermolecular and Surface Forces*. (Academic Press, 2011). doi:10.1016/B978-0-12-375182-9.10025-9

11. Timoshenko, S. & Goodier, J. N. *Theory of elasticity*. (McGraw-Hill, 1951).

12. Hiemenz, P. C. & Rajagopalan, R. *Principles of colloid and surface chemistry*. (Marcel Dekker, New York, 1986).

13. Edwards, R. L., Coles, G. & Sharpe, W. N. Comparison of Tensile and Bulge Tests for Thin-Film Silicon Nitride. *Exp. Mech.* **44,** 49–54 (2004).

14. Stachewicz, U., Bailey, R. J., Wang, W. & Barber, A. H. Size dependent mechanical properties of electrospun polymer fibers from a composite structure. *Polymer (Guildf).* **53,** 5132–5137 (2012).

15. Fromageau, J., Brusseau, E., Vray, D., Gimenez, G. & Delachartre, P. Characterization of PVA Cryogel for Intravascular Ultrasound Elasticity Imaging. *IEEE Trans. Ultrason. Ferroelectr. Freq. Control* **50,** 1318–1324 (2003).

16. Thomas, D. & Cebe, P. Self-nucleation and crystallization of polyvinyl alcohol. *J. Therm. Anal. Calorim.* **127,** 885–894 (2017).

17. Lim, C. T., Tan, E. P. S. & Ng, S. Y. Effects of crystalline morphology on the tensile properties of electrospun polymer nanofibers. *Appl. Phys. Lett.* **92,** 0–3 (2008).

18. Peresin, M. S., Habibi, Y., Zoppe, J. O., Pawlak, J. J. & Rojas, O. J. Nanofiber composites of polyvinyl alcohol and cellulose nanocrystals: Manufacture and characterization. *Biomacromolecules* **11,** 674–681 (2010).

19. Huan, S., Bai, L., Cheng, W. & Han, G. Manufacture of electrospun all-aqueous poly(vinyl alcohol)/cellulose nanocrystal composite nanofibrous mats with enhanced properties through controlling fibers arrangement and microstructure. *Polym. (United Kingdom)* **92,** 25–35 (2016).

20. Bao, C., Guo, Y., Song, L. & Hu, Y. Poly(vinyl alcohol) nanocomposites based on graphene and graphite oxide: a comparative investigation of property and mechanism. *J. Mater. Chem.* **21,** 13942 (2011).

21. Fortunati, E. *et al.* Cellulose nanocrystals extracted from okra fibers in PVA nanocomposites. *J. Appl. Polym. Sci.* **128,** 3220–3230 (2013).

22. Kondo, T., Sawatari, C., Manley, R. S. J. & Gray, D. G. Characterization of hydrogen bonding in cellulose-synthetic polymer blend systems with regioselectively substituted methylcellulose. *Macromolecules* **27,** 210–215 (1994).

23. Wingert, M. C., Chen, Z. C. Y., Kwon, S., Xiang, J. & Chen, R. Ultra-sensitive thermal conductance measurement of one-dimensional nanostructures enhanced by differential bridge. *Rev. Sci. Instrum.* **83,** 024901 (2012).

24. Weathers, A., Bi, K., Pettes, M. T. & Shi, L. Reexamination of thermal transport measurements of a low-thermal conductance nanowire with a suspended micro-device. *Rev. Sci. Instrum.* **84,** 084903 (2013).

25. Zeng, X. *et al.* Structure-induced variation of thermal conductivity in epoxy resin fibers. *Nanoscale* **9,** 10585–10589 (2017).

26. Roohani, M. *et al.* Cellulose whiskers reinforced polyvinyl alcohol copolymers nanocomposites. *Eur. Polym. J.* **44,** 2489–2498 (2008).

27. Uddin, A. J., Araki, J. & Gotoh, Y. Toward ‘Strong’ sreen nanocomposites: Polyvinyl alcohol reinforced with extremely oriented cellulose whiskers. *Biomacromolecules* **12,** 617–624 (2011).
